# When Venom Meets the Heart: A Rare Case of Scorpion Sting-Induced Acute Myocardial Infarction

**DOI:** 10.7759/cureus.44886

**Published:** 2023-09-08

**Authors:** Preethy Koshy, Gajanan Chavan, Charuta Gadkari, Shubham Dubey

**Affiliations:** 1 Emergency Medicine, Jawaharlal Nehru Medical College, Datta Meghe Institute of Higher Education and Research, Wardha, IND; 2 Nephrology, Jawaharlal Nehru Medical College, Datta Meghe Institute of Higher Education and Research, Wardha, IND

**Keywords:** ecg changes, cardiac markers, alpha blocker, autonomic dysfunction, acute myocardial infarction, scorpion sting

## Abstract

Scorpion sting cases are everyday encounters in the Emergency Department (ED). However, scorpion sting-induced systemic manifestations are rarely seen. Signs and symptoms of envenomation involve the central nervous system, stimulation of the autonomic nervous system and rarely respiratory and heart failure leading to death. Cardiovascular manifestations are particularly prominent following stings by the Indian red scorpion. This case report is of an 18-year-old male patient who presented to ED with complaints of scorpion sting. Twelve lead electrocardiography (ECG) done was suggestive of acute inferior wall myocardial infarction with raised cardiac markers. He also had autonomic dysfunction in the form of hypertension, hypothermia and priapism. He was treated with an alpha-blocker, dual antiplatelets and analgesics. ECG changes reverted to normal the next day, and he was discharged. So, the anticipation of life-threatening complications of scorpion stings plays a vital role in the treatment and prognosis of patients presenting to ED.

## Introduction

Scorpions belong to the class Arachnida and order Scorpiones [1]. This arthropod comprises 16 families and over 1500 species [2,3]. The venom comprises mucopolysaccharides, hyaluronidase, serotonin, histamine, phospholipase and neurotoxic peptides [4,5]. Clinical signs of envenomation involve the overactivity of the autonomic and central nervous system and may even cause respiratory and cardiac failure causing fatality [6]. Some of the most common presentations of scorpion envenomation include sweating, nausea, vomiting, retention of urine, salivation, priapism and pain at the bite site. The other clinical signs include hypertension, hypotension, tachycardia and arrhythmia [7]. Life-threatening complications like pulmonary oedema and myocardial infarction are also rarely seen. In severe cases, neurological complications like confusion, lethargy, agitation, impaired level of consciousness, coma and seizures may be observed [8]. Mortality is usually due to cardiac dysfunction and pulmonary oedema [9]. Cardiovascular complications are particularly seen following stings by the Indian red scorpion [10]. Such bites infrequently have severe clinical sequelae, including myocardial infarction, cardiogenic shock, acute pulmonary oedema and even death [11]. This case report is about a rare manifestation of a scorpion sting in an 18-year-old boy who presented to the Emergency Department (ED).

## Case presentation

**Figure 1:**
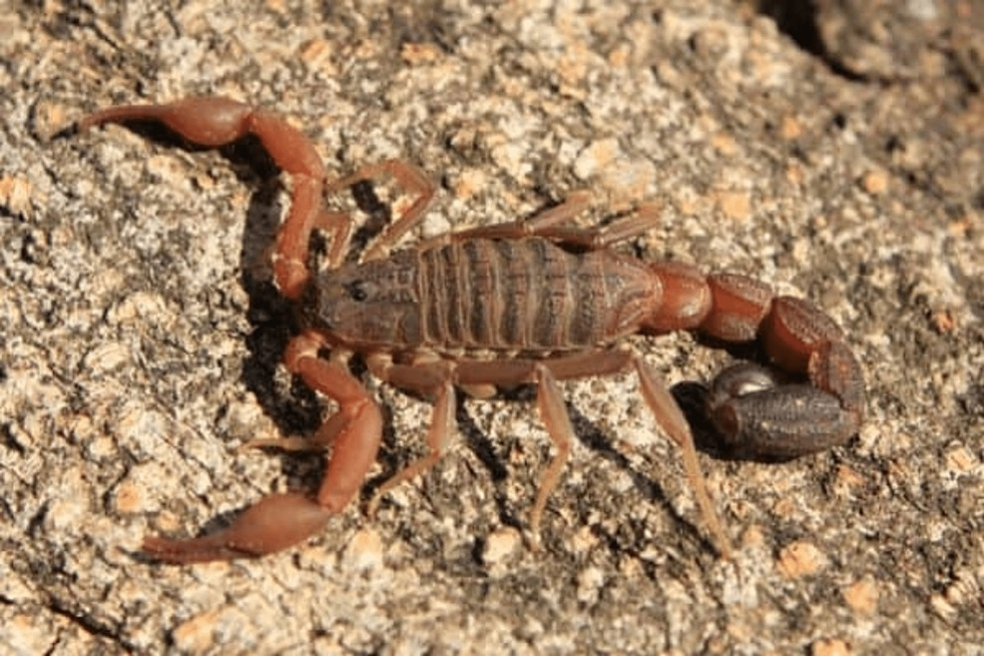
The Indian red scorpion which stung the patient.

An 18-year-old boy with no known co-morbidities was brought to the Department of Emergency Medicine of our hospital by his friend with an alleged history of scorpion sting on the distal end of his right middle finger around two hours ago at his home. Figure 1 shows the image of the Indian red scorpion.

He complained of severe aching pain extending from the right middle finger up to the right shoulder from the time of the sting. It was associated with diaphoresis and piloerection all over the body. He complained of two episodes of vomiting which were non-projectile, non-bilious, non-blood stained and contained food particles. He was taken to a nearby hospital where the Tetanus vaccine was given and was referred to our hospital for further management.

**Figure 2:**
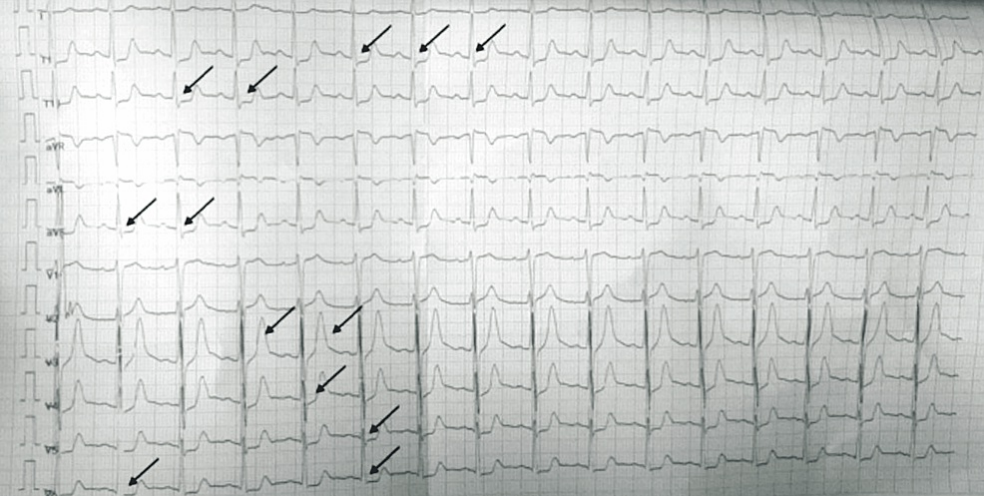
ECG of the patient showing ST depression in II, III, aVf, V3-V6 and tall T waves in V3, V4. ECG: electrocardiography

On primary assessment, he was very restless due to severe pain. Respiratory rate was 24/min maintaining an oxygen saturation of 98% on room air. The pulse rate was 62/minute with a blood pressure of 170/120 mm of Hg over the right brachial artery. He was conscious and oriented but irritable. Pupils were bilaterally equal and reactive to light. He was hypothermic with a body temperature of 34.7 degrees Celsius with severe diaphoresis and piloerection all over the body. Priapism was also noted. His systemic exams were unremarkable. Local examination of the right upper limb revealed a single sting mark on the medial aspect of the distal end of the right middle finger with oedema and tenderness up to the wrist. A 12-lead electrocardiogram taken showed ST segment depression in leads two, three, aVf and V3-V6 and tall T waves in V3, V4 as shown in Figure 2.

**Figure 3:**
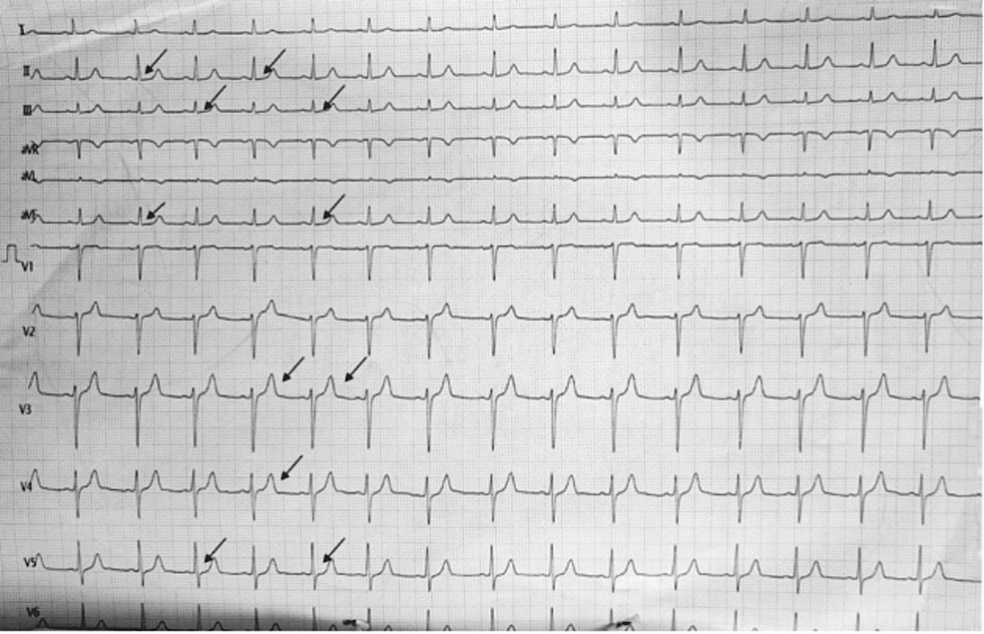
ST depression in II, III, aVf, V3-V5 and tall T waves in V3 and V4 reverted to normal the next day.

Blood reports revealed a haemoglobin of 18.6 with a haematocrit of 53.5. The total leukocyte count was raised to 22200 (neutrophils 80, lymphocytes 15). Activated partial thromboplastin time was elevated (43.2). Cardiac markers were also elevated (CKMB 63 and Troponin I 164), with total creatine kinase of 1062 and lactate dehydrogenase (LDH) of 502. Liver and renal function tests were within normal limits. He was managed in the ED with an oral alpha-blocker, dual antiplatelets, warming blankets, intravenous analgesics, a ring block with a local anaesthetic and other supportive measures. His blood pressure was continuously monitored, which eventually lowered to 140/90 mm of Hg. He was then disposed to the intensive care unit after attaining adequate analgesia. A 2D electrocardiography (ECG) showed mild left ventricular apical hypokinesia, with an ejection fraction of 55%. He was started on low molecular weight heparin, antiplatelets and antibiotics. ECG reverted to normal on the next day as shown in Figure 3.

He was advised coronary angiogram, but the patient was not willing for the same, hence, he was discharged against medical advice.

## Discussion

The scorpion venom contains nephrotoxin, neurotoxin, cardiotoxin, hyaluronidases phosphodiesterases, phospholipase, histamine and other chemicals [12]. Several pathogenetic mechanisms are involved in myocardial damage caused by the venom [13]. Various proinflammatory cytokines and vasoactive substances are present in the venom which cause damage to the blood vessels and heart. Several studies have shown a direct effect of toxins on the heart [14]. The increase in blood levels of angiotensin and catecholamines results in cardiac stimulation, severe vasoconstriction, increased oxygen requirement of the myocardium and significant changes in myocardial metabolism and perfusion [15] which may be the cause of acute myocardial infarction seen in our patient. It may also be due to sympathetic stimulation or vasospasm. The complex symptoms of scorpion envenomation may also be due to autonomic hyperactivity [5] which is seen in our patient in the form of hypertension, profuse sweating, hypothermia and priapism. The neurotoxic peptides contained in the venom interact with various ion channels and may cause severe damage to the nervous system [16]. The venoms have proven to be selective antagonists for various voltage-gated ion channels like Na+, K+ and Ca2+ channels [16-18]. These lead to membrane instability, peripheral and central nervous system blockage or dysfunction of smooth or skeletal muscle activity leading to their damage [19], which may be the reason for raised creatinine kinase in the patient. The T helper cells are responsible for inflammatory mediators against intra- and extra-cellular pathogens [20]. The balance between anti- and pro-inflammatory activities determines the extent of inflammation and thus can lead to different clinical manifestations [21-23]. Venom leads to acute lung injury leading to pulmonary inflammation and dysfunction of normal lung mechanisms. Alveolar capillary endothelial dysfunction results in interstitial and alveolar oedema [24]. However, this clinical feature was not observed in our patient. The scorpion venom also causes renal edema which leads to a decrease in glomerular filtration rate and urinary flow [25,26]. Thus, cardiovascular failure along with alveolar oedema as well as respiratory arrest may be the cause of fatality after scorpion envenomation. The direct action of the venom on the liver leading to hepatic failure may be the cause of the increment in aspartate aminotransferase (AST) levels. An increase in circulating enzyme levels like creatine phosphokinase succinate dehydrogenase and LDH has also been seen, as in our patient.

## Conclusions

Scorpion sting cases are common encounters in ED. However, scorpion sting-induced systemic manifestations are rare, even in literature. We hereby report such a rare manifestation of scorpion sting involving the cardiovascular and autonomic nervous systems. Anticipation of acute life-threatening complications like myocardial infarction and pulmonary oedema is necessary to prevent adverse events. Routine ECG, vital monitoring and clinical acumen play a great role in early detection, prevention and treatment of complications. Despite the rarity of such an association, healthcare providers in regions endemic to scorpions should be vigilant in considering myocardial infarction as a potential complication following scorpion envenomation.
